# Long-term aspirin use for cancer primary prevention: A protocol for updated systematic review and subgroup meta-analysis of randomized clinical trials

**DOI:** 10.1097/MD.0000000000017382

**Published:** 2019-09-27

**Authors:** Qibiao Wu, Hongwei Chen, Xiaojun Yao, Ting Li, Cong Xu, Jue Wang, Xinbing Sui, Elaine Lai-Han Leung

**Affiliations:** a State Key Laboratory of Quality Research in Chinese Medicine; b Faculty of Chinese Medicine; c Faculty of Medicine, Macau University of Science and Technology, Macau; d Department of Medical Oncology, Holistic Integrative Oncology Institutes and Holistic Integrative Cancer Center of Traditional Chinese and Western Medicine, the Affiliated Hospital of Hangzhou Normal University; e Department of Cancer Pharmacology, Holistic Integrative Pharmacy Institutes, College of Medicine, Hangzhou Normal University, Hangzhou, Zhejiang, China.

**Keywords:** aspirin, cancer, meta-analysis, primary prevention, randomized controlled trial, subgroup analysis, systematic review

## Abstract

**Background:**

Long-term use of aspirin for primary prevention of cancer remains inconclusive, and variation in the effects of aspirin use on cancer outcomes by cancer site, aspirin dose, follow-up duration, or different populations has never been systematically evaluated.

**Methods:**

Seven electronic databases (PubMed, EMBASE, ClinicalTrials.gov, etc) will be searched from inception to September 30, 2019. Randomized clinical trials (RCTs) comparing aspirin versus no aspirin in participants without pre-existing cancer and reporting cancer incidence, and/or cancer mortality outcomes will be selected and assessed for inclusion. The Cochrane's Risk of Bias Tool and the Jadad scale will be used to evaluate the risk of bias and the methodologic quality of the RCTs. Data will be screened and extracted by independent investigators. Total cancer incidence will be defined as the primary clinical endpoint, and total cancer mortality, all-cause mortality, and the risk of major bleeding will be the secondary outcomes. Subgroup analyses based on cancer site, aspirin dose, follow-up duration, or different populations will be conducted. Analyses will be performed using Review Manager 5.3, Comprehensive Meta-Analysis 2.0, and Trial Sequential Analysis (TSA) software.

**Results:**

This study will systematically evaluate the effects of long-term aspirin use on total cancer incidence, cancer mortality, all-cause mortality, and the risk of major bleeding. Subgroup analyses will indicate whether the effects of aspirin on cancer outcomes are associated with cancer site, daily dose of aspirin, follow-up duration, or different subgroup of participants. The results will be submitted and published in a peer-reviewed scientific journal.

**Conclusions:**

This systematic review will systematically evaluate the efficacy and safety of long-term use of aspirin for primary prevention of cancer and determine whether there are some potential influencing factors affecting the effects of aspirin on cancer outcomes, thus strengthening the evidence base for the clinical practice and future research of this intervention.

## 1 Introduction

Worldwide, cancer incidence and mortality have been rapidly increasing, and in this century, cancer is expected to become the leading cause of death.^[1–3]^ According to the World Health Organization estimation, 30% to 50% of cancer cases are preventable; how to prevent cancer and reduce cancer-related mortality has long been a major concern and there are some unquestioned prevention strategies, like reducing risk factors, adopting healthy lifestyles, and so on.^[4]^ However, there are also some controversial interventions, such as long-term aspirin use for primary prevention of cancer.

Some published trials and meta-analyses indicated that long-term use of aspirin was associated with the reduction in cancer incidence and/or cancer-related deaths,^[5–7]^ but some found no overall association between them.^[8,9]^ Also, a few studies, including 2 high-quality randomized clinical trials (RCTs) published in 2018—aspirin to reduce risk of initial vascular events (ARRIVE) and aspirin in reducing events in the elderly (ASPREE) trials—demonstrated increased cancer incidence and cancer-related mortality with aspirin.^[10,11]^ The results of published studies are conflicting and the effect of aspirin on primary prevention of cancer remains unclear.

There are a few previous meta-analyses on the role of aspirin use in cancer primary prevention, but most of them included observational trials or cohort studies which may weaken the strength of evidence compared with RCTs.^[7,12,13]^ Some only focused on 1 certain cancer,^[14,15]^ or included only subjects of specific populations, such as cardiovascular disease primary prevention populations,^[8,16]^ or only evaluate the effects of the low dose of aspirin.^[16,17]^

Aspirin's effects on total cancer incidence and mortality have not been clearly established, and subgroup analyses based on cancer site, aspirin dose, follow-up period, and the different study populations, and so on, have never been comprehensively conducted.^[8,16]^ The U.S. Preventive Services Task Force (USPSTF) emphasized the need for more research into the effects of long-term aspirin use on overall cancer, according to various aspirin doses and by subgroups, including patient characteristics, baseline cancer risk, or comorbid conditions, and so on.^[16]^

This updated meta-analysis will include more eligible RCTs, especially the new studies, to further evaluate the efficacy and safety of long-term aspirin use for cancer primary prevention, and determine the possible variation in the effect of aspirin use on total cancer incidence, cancer mortality, all-cause mortality by cancer site, aspirin dose, follow-up duration, or patient characteristics, and so on.

## 2 Materials and methods

We will perform this systematic review and subgroup meta-analysis following the PRISMA (Preferred Reported Items for Systematic Review and Meta-analysis) guidelines (Moher et al, 2009^[18]^). This study has been registered as PROSPERO (International prospective register of systematic reviews) CRD42019134083 (https://www.crd.york.ac.uk/prospero/display_record.php?RecordID=134083). Because all the materials are published articles, ethical approval is not necessary.

### 2.1 Types of studies

All RCTs comparing aspirin versus no aspirin (defined as placebo or no treatment), and reporting cancer incidence and/or cancer deaths as outcomes will be selected and assessed for inclusion in our research.

### 2.2 Types of participants

#### 2.2.1 Inclusion criteria

The trials included in this study should meet the following criteria: were RCTs; enrolled participants without known pre-existing cancer (primary prevention of cancer); compared aspirin at any dose with no aspirin; had a follow-up of more than 1 year; reported cancer incidence and/or cancer deaths as outcomes.

#### 2.2.2 Exclusion criteria

Studies on secondary or tertiary prevention of cancer, treatment of cancer, cancer remission, cancer recurrence or cancer metastases; studies where the participants were nonhuman populations, aged <18 years, pregnant women, institutionalized individuals or postsurgical patients; studies of high-incidence familial cancer syndromes (eg, Lynch syndrome); the trials were not RCTs; unavailable full-text article or unextractable data.

### 2.3 Types of Interventions

In the experimental group: aspirin; in the control group: no aspirin (placebo or no treatment).

### 2.4 Types of outcome measures

Cancer incidence is defined as the primary clinical endpoint. Cancer mortality, all-cause mortality, and major bleeding are the secondary outcomes.

### 2.5 Information sources

Three independent reviewers (Q.B.W., H.W.C., and X.J.Y.) will perform a comprehensive search of the PubMed, Embase, ClinicalTrials.gov, Anzctr.org.au, the Cochrane Library, Google Scholar, and Sciencedirect without restriction on language or publication period. A conventional search will also be performed to find potential studies that are not indexed in the electronic databases. The last search date will be September 30, 2019.

### 2.6 Search strategy

The search details will be conducted as follows: (((“aspirin”[MeSH Terms] OR “aspirin”[All Fields]) OR acetylsalicylic acid[Title/Abstract]) AND ((“carcinoma”[MeSH Terms] OR “carcinoma”[All Fields]) OR (“neoplasms”[MeSH Terms] OR “neoplasms”[All Fields] OR “cancer”[All Fields]) OR (“neoplasms”[MeSH Terms] OR “neoplasms”[All Fields] OR “neoplasm”[All Fields]) OR (“neoplasms”[MeSH Terms] OR “neoplasms”[All Fields] OR “malignancy”[All Fields]) OR (“tumour”[All Fields] OR “neoplasms”[MeSH Terms] OR “neoplasms”[All Fields] OR “tumor”[All Fields])) AND “humans”[MeSH Terms]) AND (Randomized Controlled Trial[ptyp] AND “humans”[MeSH Terms]). Furthermore, the reference lists of all the related articles will be reviewed to identify potential RCTs. There are no trials excluded due to their publication status or language.

### 2.7 Study selection

All the candidate articles will be screened by 2 independent investigators (Q.B.W. and H.W.C.) on the basis of title and abstract. The full texts will be retrieved for further evaluation according to the inclusion and exclusion criteria. All inclusion disagreements will be resolved by consensus.

### 2.8 Data extraction

Two investigators (Q.B.W. and H.W.C.) will independently rate the included RCTs and extract the data. The intention-to-treat (ITT) analysis will be used to analyze the results whenever available.

We will summarize the characteristics of all included RCTs in a table, and perform a meta-analysis using the the Review Manager (RM) 5.3 (Copenhagen: The Nordic Cochrane Centre, The Cochrane Collaboration, 2014) and the Comprehensive Meta-Analysis (CMA) 3.0 (Biostat, Englewood, NJ; 2016) software to assess the effects of aspirin on the cancer outcomes.

### 2.9 Risk of bias in individual trials

Two independent reviewers (Q.B.W. and H.W.C.) appraised the risk of bias in the included trials using the Cochrane Risk of Bias Tool for Randomized Controlled Trials.^[19]^ The following criteria will be used to evaluate bias in each trial: random sequence generation; concealment of allocation; blinding of participants and personnel; blinding of outcome assessment; incomplete data; selective reporting; and other bias. The risk of bias will be classified as ‘low,’ ‘high,’ or ‘unclear.’ The Jadad scale will also be used to evaluate the quality of the included trials, and a trial will be considered “high quality” if its Jadad score reaches 3 or greater.^[20]^

If there are disagreements, a third reviewer (E.L.L.) independently will repeat the extraction, analysis, and interpretation of the data, and the disagreements will be solved by discussion until a consensus is reached.

### 2.10 Summary measures and data synthesis

All analyses will be performed using the RM 5.3, CMA 3.0 and the Trial Sequential Analysis (TSA) software (Copenhagen Trial Unit, Centre for Clinical Intervention Research, Copenhagen, Denmark; 2011). Dichotomous data will be shown as the risk ratio (RR) or odds ratio (OR), and continuous data will be shown as the weighted mean difference (WMD) or standardized mean difference (SMD) with a 95% confidence intervals (CIs). Heterogeneity among the studies will be assessed using the *I*^2^ test. Substantial heterogeneity will be considered when *I*^2^ >50%, a random-effects model will be used (Review Manager version 5, Cochrane Collaboration, Copenhagen, Denmark) to estimate the summary RR (or OR), WMD (or SMD), and 95% CI; otherwise, a random-effects model will be applied.^[19,21–24]^ If quantitative synthesis is not appropriate, we will provide a systematic narrative synthesis with the information presented to summarize and explain the features and findings of the included RCTs.^[25]^ The Grading of Recommendations Assessment, Development and Evaluation Working Group Methodology (GRADE) will be used to assess the strength of the body of evidence.^[26]^

### 2.11 Risk of bias across trials

Egger test and funnel plots will be applied to examine the potential bias in the RCTs included in the meta-analysis when the number of RCTs is ≥10.^[19,27]^

### 2.12 Additional analyses

The TSA software, sensitivity analysis, and subgroup analysis will be applied to assess the robustness of the results and calculate the required information size in the meta-analysis.^[28]^ We will also perform a meta-regression analysis to examine the potential heterogeneity and the impact of the moderator variables on the study effect size.

## 3 Discussion

This updated systematic review and meta-analysis will include more relevant RCTs and participants to evaluate the efficacy and safety of long-term aspirin use for primary prevention of cancer; furthermore, it is the first comprehensive subgroup meta-analysis of this intervention for cancer primary prevention based on cancer site, aspirin dose, follow-up duration, and patient characteristics. The results of this systematic review will present a summary of the most present evidence on the effects of long-term use of aspirin for primary prevention of cancer, thus strengthening the evidence base for the clinical practice and future research of this intervention.

## Author contributions

**Conceptualization:** Qibiao Wu, Xinbing Sui, Elaine Lai-Han Leung.

**Data curation:** Qibiao Wu, Hongwei Chen, Xiaojun Yao, Ting Li, Jue Wang.

**Formal analysis:** Qibiao Wu, Hongwei Chen, Xiaojun Yao, Cong Xu.

**Funding acquisition:** Qibiao Wu, Xinbing Sui, Elaine Lai-Han Leung.

**Investigation:** Qibiao Wu, Hongwei Chen, Xiaojun Yao, Ting Li, Cong Xu, Jue Wang, Xinbing Sui.

**Methodology:** Qibiao Wu, Hongwei Chen, Xiaojun Yao, Ting Li, Jue Wang.

**Project administration:** Qibiao Wu, Xinbing Sui, Elaine Lai-Han Leung.

**Resources:** Qibiao Wu.

**Software:** Qibiao Wu, Hongwei Chen, Xiaojun Yao, Ting Li, Jue Wang.

**Supervision:** Qibiao wu, Elaine Lai-Han Leung.

**Validation:** Qibiao Wu, Ting Li, Cong Xu, Xinbing Sui, Elaine Lai-Han Leung.

**Visualization:** Qibiao Wu.

**Writing – original draft:** Qibiao Wu, Hongwei Chen.

**Writing – review & editing:** Qibiao Wu, Xinbing Sui, Elaine Lai-Han Leung.

QIBIAO WU orcid: 0000-0002-1670-1050.
